# Experience with 224 percutaneous dilatational tracheostomies at an adult intensive care unit in Bahrain: A descriptive study

**DOI:** 10.4103/1817-1737.37949

**Published:** 2008

**Authors:** Akmal A. Hameed, Hasan Mohamed, Mariam Al-Ansari

**Affiliations:** *Department of ICU, Salmaniya Medical Complex, Manama, Kingdom of Bahrain*

**Keywords:** Complication, percutaneous dilatational tracheostomy, ventilation, weaning

## Abstract

Tracheostomy is one of the most commonly performed procedures in critically ill patients. Over the past 15 years, many large university hospitals have reported their experience with percutaneous dilatational tracheostomy (PDT). We have described and compared our experience with 224 PDTs that we performed in the last four and a half years. We have also compared PDT performed with and without bronchoscopic guidance at our setting and PDT verses surgical tracheostomy.

**OBJECTIVE:**

The purposes of this study are to evaluate the safety of PDT in our hospital setting and to compare our results with those published in the literature.

**DESIGN:**

A retrospective study for our experiences about safety and efficacy of 224 PDTs in an intensive care unit (ICU) setting.

**SETTING:**

A 11-bedded adult medical, surgical, neuro-trauma ICU at Salmaniya Medical Complex, Bahrain.

**MATERIALS AND METHODS:**

This is based on our experiences about complications/timings of all PDTs performed from October 2002 to February 2007. A retrospective chart analysis.

**RESULTS:**

There were 15 mechanical complications in total, including nine patients developing bleeding during or post-procedure, three patients developing pneumothorax and two patients developing cellulitis; in one procedure, a tracheostomy tube was misplaced. The proportion of total complications was 6.6% and no death.

**CONCLUSIONS:**

From our experience, bronchoscope can be used during PDTs performed in ICU by inexperienced intensivists who do not have good exposure to procedures, but after gaining adequate experience, PDT can be performed safely without using bronchoscope.

## Introduction

Tracheostomy, an ancient surgical procedure originally described in the first century BC,[1] is one of the most commonly performed procedures in modern intensive care and is predicted to become more common as demand for intensive care services increases.[2] The correct time for tracheostomy is still not clear, while the benefits of early tracheostomy in patients who require extended periods of mechanical ventilation, as compared to prolonged translaryngeal intubation, have been recently debated.[3–5] The optimal method of performing tracheostomies in critically ill patients remains unclear.

Percutaneous dilatational tracheostomy (PDT) was first described in 1957,[6] and became increasingly popular after the release of a commercially available kit in 1985.[7] This technique involves the use of blunt dilatation to open the pretracheal tissue for passage of the tracheostomy tube. Proponents of PDT suggest that limited dissection results in less tissue damage, lowers the risk of bleeding and wound infection and is able to be performed at bedside in the ICU, which may overcome the risks associated with transport of critically ill patients to the operating theatre.[8]

The purposes of this study are to evaluate the safety of PDT in our hospital setting and to compare our results with those published in the literature

In patients on whom we performed PDT, the indications for tracheostomy included the following: difficult weaning process (mostly patients with critical illness neuropathy and myopathy and some patients with pre-existing pulmonary diseases), cerebral injury (usually severe traumatic head injury, postanoxic injury or cerebral infarction) and other neurological disorders (e.g., spinal cord injury and Guillain-Barre syndrome).

## Materials and Methods

The Salmaniya Medical Complex, Bahrain, is one thousand-bedded hospital with a 11-bedded ICU. The average admission of patients to ICU was 45 to 50 per month. The average acute physiological and chronic health evaluation (APACHE II) of patients in the last 5 years was 22.6 and mortality rate was 11.2%. From October 2002 to February 2007, all intubated patients who required prolonged ventilation and had difficulty in weaning from ventilators were assessed. The indications for PDT included the following: anticipated need for long-term airway access (protection) and artificial airway access needed for prolonged mechanical ventilation. Written consent from all patients was obtained for the purpose of the procedure. Patients with short fat neck, cervical fracture and ‘uncleared’ cervical spine or had difficulty in intubation were referred for open surgical tracheostomy (ST). All PDTs were performed in the ICU. In over a four-and-a-half year's period, 224 PDTs were performed, and 39 patients who required tracheostomy were referred to an ENT surgeon due to short neck or difficulty in intubation and performed surgery in an operating room (OR).

**Inclusion criteria:** All adult patients who were admitted to ICU and required mechanical ventilation and anticipated prolonged ventilation.

**Exclusion criteria:** Patients with abnormal anatomical deformities in neck or history of difficulty in intubation were referred for surgical tracheostomy.

**Type of set:** Kit (Portex; Hythe, Kent, UK) with curved dilating forceps.

## Results

Data were collected as part of day-to-day ICU auditing system including APACHE II. All PDTs were done in the ICU by a consultant intensivist. All STs were performed in an OR. 

A total of 224 PDTs were performed in the last four and a half years in this 11-bedded ICU. One hundred and sixteen (51.8%) male and 108 (48.2%) female patients underwent this procedure [Table 1]. One hundred and eighty-one (80%) patients were primarily admitted in medical faculty, 26 (11.6%) patients in surgery and 17 (7.59%) patients in neuro-trauma [Table 1]. There were 15 complications and no death [Table 2]. No procedure-related fatalities occurred during PDT. The correct position of tracheal cannulation (which is defined as a secured position in midline of the trachea) was achieved in all patients (100%). There was one paratracheal and three pneumothorax insertions, and no tracheal tear was observed [Table 2]. A comparison of our results with those reported in five recently published articles in literature showed no significant difference in mortality rate, pneumothorax, bleeding, paratracheal placement, dislodgement or cellulites [Table 3]. There was a trend of significantly lower incidence of paratracheal placement using bronchoscopic guidance. We used bronchoscopic guidance in the initial stage for first 52 PDTs performed,[9] but we did not find chances of complications being lesser in using bronchoscopic guidance [Table 4].

**Table 1:** Patient population

	No. of patients (224)	Age	
		
		Medien	Range
Male	116 (52)	-	-
Female	108 (48)	-	-
Trauma	17 (7.59)	41	18–71
Medical	181 (80)	70	28–89
Surgical	26 (11.6)	55	22–66
PDTs with bronchosope	52 (23.2)	59	25–78

Figures in the parentheses are in percentage

**Table 2:** Noted complications in percutaneous dilatational tracheostomys performed

Complication	No. of patients	Percentage of total
Death	0	0
Pneumothorax	3	1.33
Tracheal tear	0	0
Bleeding	9	4.0
Cellulitis	2	0.89
Misplacement	1	0.44
Total no of complications	15	6.6

**Table 3:** Comparison of our experience with other published studies[13–15]

Complications	Studies					
	
	Ciaglia and Graniero 165	Cobean *et al.* 65	Hill *et al.* 353	Barba *et al.* 27	Walz *et al.* 326	Our study 224
No. of deaths	0	1	1	1	2	0
Pneumothorax	2	2	2	0	0	3
Tracheal tear	0	0	1	0	0	3
Bleeding	3	3	9	0	2	7
Cellulitis	1	0	2	0	4	2
Dislodgement	0	1	6	0	6	0
Misplacement	1	1	6	0	2	1
No. of deaths	0	1	1	1	2	0

**Table 4:** Comparison of percutaneous dilatational tracheostomys performed with and without bronchoscopic guidance

Complications	52 PDTs with bronchoscope	172 PDTs wihtout bronchoscope	*P*-value
Death	0	0	Not compatible
Pneumothorax	1 (1.9%)	2 (1.1%)	0.0001
Tracheal tear	0	0	Not compatible
Bleeding	4 (7.6%)	5 (2.9%)	0.2229
Air way loss	0	0	Not compatible
Cellulitis	1 (1.9%)	1 (0.44%)	0.0001
Misplacement	0	1 (0.44%)	Not compatible
Total	6 (11.5%)	9 (4.01%)	0.6343

*P*-values at significant level of 95%, PDTs - Percutaneous dilatational tracheostomys

### Statistical analysis

Data were analysed with Medcalc version 9.3. The procedure used to calculate *P*-value was goodness-of-fit Chi-square test.

## Discussion

Tracheostomy has been done in chronically ventilated patients for over 30 years. The procedure has multiple benefits. The patient's airway is easier to access, and the patient can be more comfortable.[10] The most convincing argument is the stability of the airway once a tracheostomy has been placed and has matured. In the mid-1980s, Ciaglia *et al.*[11–12] published their experience with PDT and showed that this new method was safe. In October 2002, we began using PDT in ICU at Salmaniya Medical Complex, Bahrain. So far, we have performed 224 PDT procedures, initially 52 using bronchoscopic guidance and later 172 without bronchoscope.

We studied the safety and short-term complications of PDT, as performed by an intensivist on patients in our ICU. All patients were carefully evaluated and followed up to assess the complications and adverse effects of PDT.

From October 2002 to February 2007, a total of 2592 patients were admitted to this ICU. The mortality rate was 11.2% with average APACHE II being 22.6. Two hundred and twenty-four PDTs were performed in the ICU. There was no death, but we reported 15 complications: in four early cases, airways were lost but were easily and immediately re-established without any consequences; nine patients had excessive bleeding after PDT, which was controlled by pressure bandage but did not require any blood transfusion. There was one incident of paratracheal placement, and in three patients who developed pneumothorax, chest tubes were inserted, which were removed after 5-6 days; two patients had developed cellulites around the site of tracheostomy tube but did not require any intervention or antibiotics for a specific reason. There were 15 complications (6.6%) out of a total of 224 PDTs performed at this ICU [Table 2]. A comparison of our results with those reported in five recently published articles[13–15] showed no significant difference in mortality rate, pneumothorax, bleeding, etc. [Table 3]. There was a trend of lower incidence of paratracheal placement using bronchoscopic guidance. We had performed the initial 52 procedures under bronchoscopic guidance, and bronchoscopic examination was done immediately after PDT to rule out any immediate complications, and the remaining 172 PDTs were performed without bronchoscopic guidance and did not find any change or reduced form of complication [Table 4]. In fact, 52 PDTs performed under bronchoscopic guidance had a total complication rate of 11.5%, whereas in 172 PDTs performed without bronchoscopic guidance, the total complication rate was 4.01% [Table 4].

Thus, the PDT insertion procedure was completely uneventful in 93.7% of patients and in 89.5% when bronchoscopic guidance was used [Table 4] and in 94.8% without bronchoscopic guidance.

The mean operative time for percutaneous tracheostomy was 20.10 (±16.10) min. The shortest duration recorded was 8 min, and the longest duration recorded was 35 min.

In our experience, the longest time incurred in bedside PDT was due to the preparatory steps, from adequate premedication to proper endotracheal tube positioning.

All our patients were critically ill; the average APACHE score of our patients was 22.6; in spite of the high severity of illness, we did not come across any major complication, which is related to primary sickness. Therefore, PDT can be performed safely in critically ill patients without any major complication in ICU.

In 1989, The American College of Chest Physicians' Consensus Conference on Artificial Airways in Patients Receiving Mechanical Ventilation suggested that translaryngeal intubation was the preferred technique for patients requiring up to 10 days of mechanical ventilation.[16] Mohr *et al.*[17] concluded that early tracheostomy facilitates weaning from mechanical ventilation, but have not specified the optimum time for tracheostomy. On the other hand, Sugerman *et al.*,[18] in their multi-center, randomized, prospective trial of early tracheostomy, have concluded that there is no significant benefit from early tracheostomy. In our experience, there was no significant difference in the outcome in terms of mortality and early weaning from ventilation in patients who underwent tracheostomy within 10 days or more than 10 days of endotracheal intubation.

PDT is clearly the procedure of choice in many situations. However, there are contraindications for PDT. It is not designed to replace cricothyroidotomy for an emergency surgical airway. It also has not been adequately studied for use in the pediatric population. It should be used with caution in patients with difficult airway and in those with unstable or presumed cervical spine injuries. Certain anatomic conditions are relative contraindications for PDT. These include an inability to palpate the cricoid cartilage, morbid obesity, thyroid goiter and deviation of the trachea. If inadvertent extubation and potential direct laryngoscopy for reintubation cannot be tolerated, then PDT should not be considered.

As far as technical aspect of procedure is concerned, several clinical trials have compared the various methods of performing PDT, but without any method being shown to be conclusively superior.[19] Since a majority of studies included in this review used the multiple dilator technique, it is not surprising to know that the results failed to demonstrate any particular benefit from one specific technique of PDT. While it has been suggested that the use of a bronchoscope to guide the operator performing the PDT makes the procedure safer,[20] this was not supported by the results of our analysis.

Our protocol for performing PDT involved the performance of bronchoscopy during and immediately following PDT to check the final position of the tracheal cannula. For this reason, we are reasonably sure that problems of edema and abrasions of the posterior tracheal wall have not developed in our patients but later we abandoned using bronchoscope and did not encounter any major complications.

Paratracheal insertion of the tracheostomy tube is a well-known complication of percutaneous tracheostomy. One (0.4%) out of the 224 patients in our study who underwent PDT procedures had paratracheal insertion of tube, and the procedure was continued and done successfully after second incision. The incidence of paratracheal insertion has been reported by various authors as 0.8%, especially by Powell *et al*.[21] One explanation given for this complication is the calcification of tracheal rings especially in elderly patients. Calcified cartilage will tend to deflect the dilators and bend the guidewire despite proper placement of needle and guidewire. As a result, the dilators and tracheostomy tube can create a false passage into the anterior mediastinum. The second important complication, which we encountered in a 60-year-old female patient during percutaneous tracheostomy, was that the patient developed bilateral pneumothorax (0.4%) and had cardiac arrest but successfully revived without any anoxic brain damage. Bilateral chest tube was inserted and the pneumothorax subsided. Chest tube was removed after 5-6 days and the patient recovered completely, and later surgical tracheostomy was done in an OR. Powell *et al.* reported 0.6% incidence of pneumothorax during PDT.[21] Cheng and Fee have described an overall incidence of pneumothorax as 1% for PDT and 4% for open.[22]

Although the technique of minimally invasive PDT is being used more and more widely in Europe and the United States, especially in ICUs, its exact role remains a matter for debate. Issues that remain controversial include whether PDT has more or fewer complications than traditional ST, how and by whom PDT should be performed, what, if any, precautions (such as bronchoscopic or ultrasound guidance) to take and which categories of patients are suitable candidates for this procedure. Two meta-analyses have come to different conclusions. Dulguerov *et al.*[23] have analysed 65 studies (38 dealing with ST and 27 dealing with PDT, published between 1960 and 1996) and concluded that PDT was associated with a higher prevalence of perioperative complications, perioperative deaths and cardiorespiratory arrests. In contrast, Freeman *et al.*[24] have concluded from a recta-analysis of five prospective controlled studies involving 236 patients that PDT was easier to perform, produced fewer overall postoperative complications, needed shorter operative times, less postoperative and perioperative bleeding and fewer postoperative stomal infections than did ST. These authors concluded that their findings support PDT as the procedure of choice for the establishment of elective tracheostomy in critically ill patients but that additional data were required.[24] This view was reinforced in an accompanying editorial that stressed the heterogeneity of outcomes in the studies used for this meta-analysis.[25] We referred 39 patients for open ST from October 2002 to February 2007, due to short neck and history of difficult intubation, and one patient was moderately obese and during ST the airway was lost, which could not be secured and the patient expired. There were eight complications with one being fatal; the total complication rate was 20.5%. One patient had developed pneumothorax and required chest tube but subsided after 5 days and chest tube was removed. One patient developed cellulitis around the tube but did not require any intervention. There was one incidence of paratracheal placement of tracheostomy tube but it was reinserted with the same incision without any complication. Three patients encountered bleeding but was controlled with pressure bandage and did not require any transfusions [Table 5]. All STs were performed by ENT consultants or senior residents in an OR. The mean operating time for ST was 35 min; the shortest duration was 30 min. The time taken to transport patients from ICU to OR and waiting outside the OR was approximately 93 min.

**Table 5:** Comparison of complications between percutaneous dilational tracheostomys verses surgical tracheostomys

Complications	PDTs (224)	Surgical tracheostomies (134)	*P*-value
Death	0	1 (0.74)	Not compatible
Pneumothorax	3 (1.3)	3 (2.2)	0.0025
Tracheal tear	0	0	Not compatible
Bleeding	9 (4)	5 (3.7)	0.1574
Airway loss	0	3 (2.2)	Not compatible
Misplacement	1 (0.44)	1 (0.74)	0.0001
Cellulitis	2 (0.89)	3 (2.2)	0.0006
Total	16 (11.9)	15 (6.6)	0.9118

Figures in the parentheses are in percentage

As far as post-PDT care is concerned, tracheal dislodgement has been the Achilles heel of all tracheostomies for decades. Dislodgement in PDT can lead to severe respiratory distress and possibly death. The newly dilated tract into the trachea usually closes within seconds if the tube was removed. Attempts at forcing a tube into this tract have been usually not successful and are ill advised. If the PDT tube gets dislodged, the patient should be orally intubated. The best way to prevent this complication is to take precautionary measures. Once the PDT is placed, we suggest suturing the tube in place and securing the tube by using umbilical tape through the eyelets of the tracheostomy tube and tying the tape around the neck. Sedation for agitated patients is paramount. Both ICU nurses and respiratory therapists must be acutely aware of this potentially fatal complication. Ventilator tubing should be secured in such a way to prevent pulling on the newly placed tracheostomy tube and thus prevent accidental dislodgement.

Bleeding is the most frequent complication in the combined data. As with all patients undergoing a surgery, it is paramount that the patient did not have a coagulopathy. One study[13] included a relatively large number of patients requiring dialysis for renal failure. Because several of these patients did have bleeding after the procedure, we recommend that the platelet count, prothrombin time and partial thromboplastin time be measured before PDT in all patients with renal failure.

The overall incidence of clinically relevant bleeding was 5.7% (*n *= 49/861) based on the data available from 10 randomized controlled trials (RCTs),[26] whereas in our study it was 4.05% (*n* = 12/222).

Clinically important wound infection was diagnosed in 6.6% (*n *= 57/870) of patients based on the data from 11 RCTs,[27] and in our study cellulites developed in only one patient (*n* = 2/224, 0.89%).

All patients undergoing PDT should have chest radiography subsequently to rule out pneumothorax and placement of tube.

## Conclusions

From our experience, bronchoscope can be used during PDTs performed in ICU by inexperienced intensivists who do not have good exposure to procedure, but after gaining adequate experience, PDT can be performed safely without using bronchoscope.

PDT is a safe and effective procedure when performed by a team of experienced physicians under controlled circumstances. The intermittent obstruction of the cannula caused by the swelling and irritation of the posterior tracheal wall should be considered in patients who may develop unexplained paroxysmal respiratory problems some time after PDT. Sudden desaturation in a patient who had just had a PDT should cause the intensivist to act quickly. Differential diagnosis would include tension, pneumothorax and misplacement or dislodgement of the tracheostomy tube. These patients should be immediately orally intubated with bilateral chest decompressions (large bore needle placed in the second intercostal space along the midclavicular line). As far as our experience is concerned, PDT is safer, less time-consuming and provides lesser complications that ST.

## References

[1] Walts PA, Murthy SC, DeCamp MM (2003). Techniques of surgical tracheostomy. Clin Chest Med.

[2] Needham DM, Bronskill SE, Calinawan JR, Sibbald WJ, Pronovost PJ, Laupacis A (2005). Projected incidence of mechanical ventilation in Ontario to 2026: Preparing for the aging baby boomers. Crit Care Med.

[3] Griffiths J, Barber VS, Morgan L, Young JD (2005). Systematic review and meta-analysis of studies of the timing of tracheostomy in adult patients undergoing artificial ventilation. BMJ.

[4] Freeman BD, Borecki IB, Coopersmith CM, Buchman TG (2005). Relationship between tracheostomy timing and duration of mechanical ventilation in critically ill patients. Crit Care Med.

[5] Nieszkowska A, Combes A, Luyt CE, Ksibi H, Trouillet JL, Gibert C (2005). Impact of tracheotomy on sedative administration, sedation level and comfort of mechanically ventilated intensive care unit patients. Crit Care Med.

[6] Shelden CH, Pudenz RH, Tichy FY (1957). Percutaneous tracheotomy. J Am Med Assoc.

[7] Ciaglia P, Firsching R, Syniec C (1985). Elective percutaneous dilatational tracheostomy: A new simple bedside procedure; preliminary report. Chest.

[8] Al-Ansari MA, Hijazi MH (2005). Clinical review: Percutaneous dilatational tracheostomy. Crit Care.

[9] Hameed AA, Mohamed H, Ghuloom AE (2004). Experience with percutaneous dilatational tracheostomy. Clin Intens Care.

[10] Walts PA, Murthy SC, DeCamp MM (2003). Techniques of surgical tracheostomy. Clin Chest Med.

[11] Grillo HC, Mathisen DJ, Sabiston DC (1997). Tracheostomy and its complications. Textbook of Surgery: The Biological Basis of Modern Surgical Practice.

[12] Ciaglia P, Firshing R, Suyniec C (1985). Elective percutaneous dilatational tracheostomy: A simple bedside procedure: Preliminary report. Chest.

[13] Hill BB, Zweng TN, Maley RH (1996). Percutaneous dilatational tracheostomy: Report of 356 cases. J Trauma.

[14] Barba CA, Angood BP, Kauder DR, Latenser B, Martin K, McGonigal MD (1995). Bronchoscopic guidance makes percutaneous dilatational tracheostomy a safe, cost-effective and easy-to-teach procedure. Surgery.

[15] Walz MK, Peitgen K, Thurauf N, Trost HA, Wolfhard U, Sander A (1998). Percutaneous dilatational tracheostomy-early results and long term outcome of 326 critically ill patients. Intensive Care Med.

[16] Plummer AL, Gracey DR (1989). Consensus conference on artificial airways in patients receiving mechanical ventilation. Chest.

[17] Mohr AM, Rutherford EJ, Cairns BA, Boysen PG (2001). The role of dead space ventilation in predicting outcome of successful weaning from mechanical ventilation. J Trauma.

[18] Sugerman HJ, Wolfe L, Pasquale MD, Rogers FB, O'Malley KF, Knudson M (1997). Multicenter, randomized, prospective trial of early tracheostomy. J Trauma Inj Infect Crit Care.

[19] Ambesh SP, Pandey CK, Srivastava S, Agarwal A, Singh DK (2002). Percutaneous tracheostomy with single dilatation technique: A prospective, randomized comparison of Ciaglia blue rhino versus Griggs' guidewire dilating forceps. Anesth Analg.

[20] Oberwalder M, Weis H, Nehoda H, Kafka-Ritsch R, Bonatti H, Prommegger R (2004). Videobronchoscopic guidance makes percutaneous dilational tracheostomy safer. Surg Endosc.

[21] Powell DM, Price PD, Forrest LA (1998). Review of percutaneous tracheostomy. Laryngoscope.

[22] Cheng E, Fee WE (2000). Dilatational versus standard tracheostomy: A meta-analysis. Ann Otol Rhinol Laryngol.

[23] Dulguerov P, Gysin C, Perneger TV, Chevrolet JC (1999). Percutaneous or surgical tracheostomy: A meta-analysis. Crit Care Med.

[24] Freeman BD, Isabella K, Lin N, Buchman TG (2000). A meta-analysis of prospective trials comparing percutaneous and surgical tracheostomy in critically ill patients. Chest.

[25] Heffner JE (2000). Percutaneous dilatational vs standard tracheostomy: A meta-analysis but not the final analysis. Chest.

[26] Massick DD, Yao S, Powell DM, Griesen D, Hobgood T, Allen JN (2001). Bedside tracheostomy in the intensive care unit: A prospective randomized trial comparing open surgical tracheostomy with endoscopically guided percutaneous dilational tracheotomy. Laryngoscope.

[27] Antonelli M, Michetti V, Di Palma A, Conti G, Pennisi MA, Arcangeli A (2005). Percutaneous translaryngeal versus surgical tracheostomy: A randomized trial with 1-yr double-blind follow-up. Crit Care Med.

